# Influence of Acidic Environmental Conditions on Push-Out Bonding Strength of Four Calcium Silicate-Based Materials to Root Dentin

**DOI:** 10.1155/2022/9169221

**Published:** 2022-09-19

**Authors:** Beliz Özel, Raif Erişen

**Affiliations:** ^1^Department of Endodontics, Yeditepe University, Istanbul, Turkey; ^2^Department of Endodontics, Istanbul Nisantasi University, Istanbul, Turkey

## Abstract

**Introduction:**

Calcium silicate-based cements (CSCs) are frequently used in various endodontic procedures such as perforation repair, vital pulp therapy, regenerative treatments, or apexification. One of their areas of use, treatment of perforations, can be challenging in clinical practice. Selection of stable, durable, and compatible material with structural and biological alterations is a must in such situations.

**Aim:**

This study aimed to compare the dislocation resistance of various calcium-silicate-containing materials used in endodontic treatment exposed to various environmental conditions in a push-out study model.

**Methods:**

Selected ninety-six human mandibular premolars with single root canals were cut from the middle portion to obtain dentin slices of 2 mm thickness (*n* = 192). Then, the canal lumen was enlarged by using #4Gates-Glidden drills. Specimens for each repair material (MTA, Angelus, Endosequence RRM (ERRM), Biodentine, BioMTA) were placed in shaped lumens, wrapped in pieces of gauze, and randomly divided into four groups (*n* = 48) according to the storage time and media: group A: 4 days in phosphate-buffered saline (PBS), group B: 4 days in acetic acid (pH = 4.4), group C: 34 days in PBS, and group D: 4 days in acetic acid (pH = 4.4) followed by exposure to PBS for 30 days. A universal testing machine measured the dislodgement resistance followed by scanning electron microscopy imaging to evaluate the material-dentin interface.

**Results:**

ERRM showed the highest dislocation resistance in all test groups (*p* < 0.05). The greatest bonding strength was observed (13,54 ± 5,56 MPa) after exposure to 34 days in PBS (pH = 7.2). The values for ERRM decreased in contact with acetic acid (pH = 4.4) and increased when placed in PBS (*p* > 0.05).

**Conclusion:**

All repair materials showed a higher dislocation resistance when stored in PBS regardless of storage time. However, the improved pH of the surrounding media was not successful in reversing the deteriorating effect caused by lower pH in relation to dislocation resistance in all tested materials except for ERRM.

## 1. Introduction

Perforations are complications that may develop during endodontic treatment, affecting the longer prognosis of the treatment. The primary reason for their occurrence is iatrogenic; however, they may also happen through resorptive processes or caries [1]. On the other hand, a furcal perforation connects the root canal system and the surrounding structures of the tooth, resulting in the loss of the periodontal attachment [2]. Therefore, an appropriate bond strength at the material-dentin interface is crucial to ensure a proper seal of the root canal system with the ultimate goal of preventing or minimizing microleakage. An ideal material for such occasions should adapt sufficiently to the canal wall, be easily manipulated, be dimensionally stable, and be biocompatible [3].

Calcium silicate-based cements (CSCs) are hydraulic self-setting materials which comprised dicalcium and tricalcium silicates providing adequate biocompatibility and bioactivity [4]. During their setting reaction, they form a calcium-silicate-hydrate (CSH) gel [5], followed by the solidification of the product into a rigid structure [6], producing an alkaline pH [7]. This setting reaction of a CSC is essential as it controls the bioactivity of these materials.

Various CSCs were preferred in many endodontic situations such as apexification, vital pulp therapy, orthograde and retrograde filling, and the repair of perforations due to their superior sealing ability, biocompatibility, and regenerative capability [8–12]. Mineral trioxide aggregate (MTA) (Angelus, Londrina, Brazil), a calcium silicate-based material, is used in diverse clinical scenarios [8]. Novel CSCs such as Biodentine (Septodont, Saint Maur des Fosses, France), with its good handling and superior biological, mechanical, and physical properties [13] or Endosequence root repair material (ERRM) (Brasseler, Savannah, GA, USA), have been shown to have superior biocompatibility and bioactivity potential [14]. More recently, a novel CSC, OrthoMTA (BioMTA, Seoul, Korea), was investigated in several studies for biocompatibility [15] and chemical composition [16]; however, its stability as a repair material is yet to be understood.

CSC demonstrated a bioactive behavior when secluded in a phosphate-containing fluid through the formation of metastable amorphous calcium-phosphate nanoparticles [17]. This physiochemical interaction with the local environment improves the adherence and biocompatibility of these materials [18, 19]. From a clinical perspective, an interfacial layer forming between the material and dentin improves the adaptation of the material [20], resulting in fluid-tight sealing of the treated area. However, alterations in surrounding tissues could affect the setting reaction, such as an acidic environment decreasing the physicochemical properties of these materials [3]. As previously reported, the pH of periapical abscesses is generally acidic [21], and such a high level of inflammation of the surrounding tissues may decrease the pH as low as 5.5 [22]. Placement of CSCs in contact with inflamed conditions can ease the dislocation of the material and increase leakage [23] due to the nonsetting interfacial area.

There have been many studies comparing the dislocation resistance of CSCs in different environmental conditions [23–25]. However, there is a lack of data comparing the bioactivity potential of newly introduced materials. Thus, this study aimed to compare the dislocation resistance of four CSC repair materials when stored in acidic pH or phosphate-buffered saline (PBS) and investigate whether storage in improved conditions could reverse the compromised effect.

## 2. Materials and Methods

**Figure 1:**
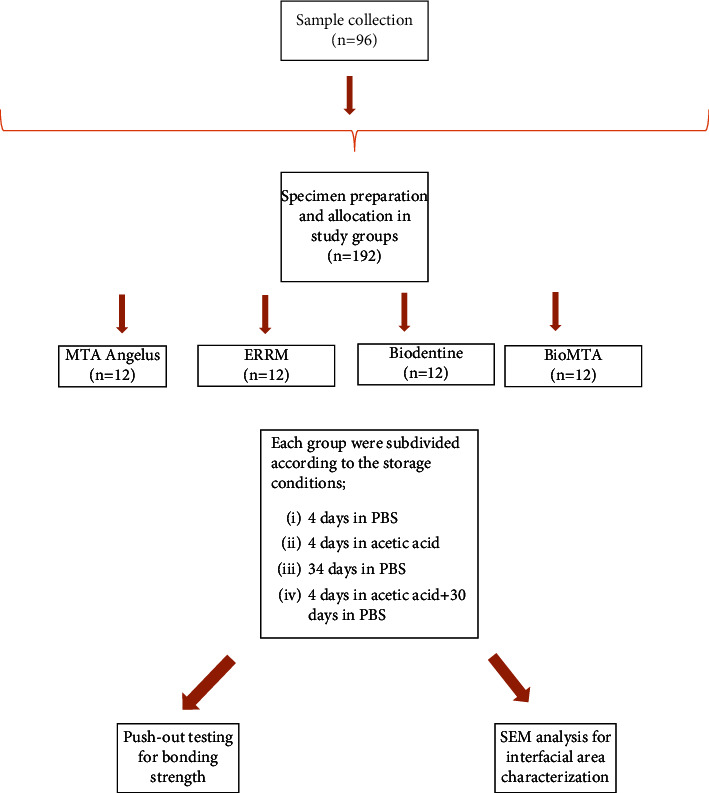
Study flow chart.

The sample size was determined with an effect size of 1.4 [23], resulting in 192 specimens (*n* = 48) to detect a difference between the groups with an *α* of 0.05 and 80% power to compare four independent proportions (see Figure1).

### 2.1. Specimen Preparation

**Figure 2:**
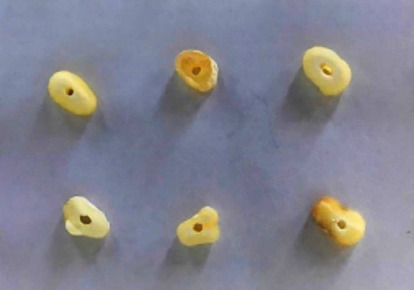
Specimens prepared for the study (lumen size 1.3 mm).

Ninety-six extracted human mandibular premolars with single root canals collected from Istanbul University Department of Surgery Clinics were examined under a stereomicroscope (Olympus, Tokyo, Japan) with 10x magnification prior to sectioning (Figure 1). The inclusion criteria were similar anatomy, straight mature roots without any resorption, and freedom from cracks or cavities. Teeth not providing these features were excluded. Radiography was taken both from buccolingual and mesiodistal directions to determine whether the canal anatomy was suitable. A# 10*K*-type file was inserted following access cavity preparation to ensure patency. The middle portion of each root was cut by using a water-cooling diamond blade microtome (ISOMET, Buehler Ltd, Lake Buff, USA) to produce 2 mm thick dentin specimens. A digital caliper (Mitutoyo, Tokyo, Japan) with a precision of 0.001 mm was used to measure the final thickness. Lastly, the lumens of the sectioned slices were enlarged by using Gates-Glidden burs size 2 – 4 to achieve a standardized diameter of 1.3 mm (Figure 2).

### 2.2. Experimental Groups

The samples were randomly allocated into four test groups (*n* = 48), MTA Angelus, ERRM, Biodentine, and BioMTA, by using a sequence generator (https://www.random.org). Subsequently, samples in each group were divided into four groups (*n* = 12) according to the storage media and time: (a) samples placed for four days in contact with a phosphate-buffered solution at pH 7.2, (b) samples placed for four days in contact with acetic acid buffered to pH 4.4, group (c) samples placed for thirty-four days in contact with PBS, and (d) samples placed for four days in contact with acetic acid buffered to pH 4.4 followed by thirty days in contact with PBS. Prepared solution of PBS included 0.17 g KH_2_PO_4_, 1.18 g Na_2_HPO_4_, 8.0 g NaCl, and 0.2 g KCl [26] with distilled water and buffered to pH 7.2 using NaOH as a basic compound [25]. In preparation of acetic acid, 1 *µ*l of acid was mixed with distilled water and buffered to pH 4.4.

**Figure 3:**
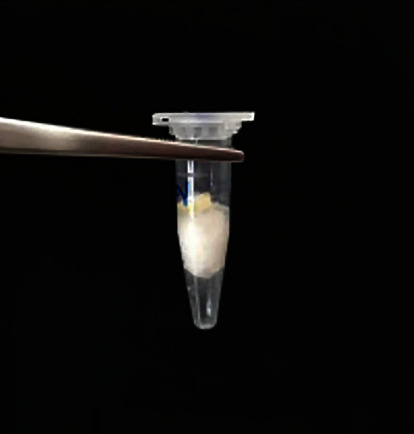
A specimen wrapped in gauze.

The materials were mixed accordingly, applied onto the lumens of slices, and condensed by using an endodontic plugger size #2–4 (Dentsply, Maillefer, Switzerland). The samples were wrapped in gauze soaked in experimental solution (Figure 3), changed every four days, and incubated at 37°C and 100% humidity.

### 2.3. Push-Out Test

**Figure 4:**
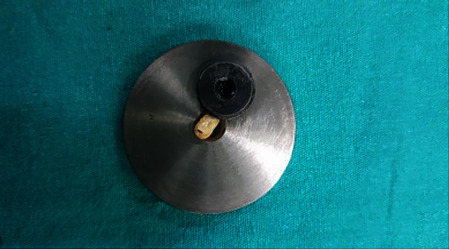
Metal base for samples to be placed on prior to push-out testing.

**Figure 5:**
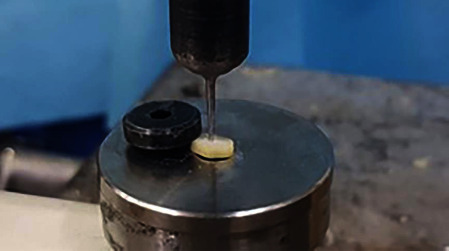
Push-out testing with a metal plunger.

Push-out bond strength measurements were carried out by using a universal testing machine (MTS, Bionix II, MN, USA). The samples were placed on a metal base (Figure 4) with a hole in the middle to free the test lead's movement. A 1.1 mm diameter stainless steel plunger (Figure 5), moving at a 1 mm/min speed, exerted a driving force on the material at a load cell of 10 kN. The tip boundaries were 0.2 mm away from the dentine margins to prevent the plunger from contacting the teeth. The material received a constant force from the test tip until complete dislocation from the prepared cavity. Dislodgement of the material was recorded as the maximum load in newtons (N). The push-out bond strength (MPa) was calculated with the following formula: MPa = N/2*πr* × *h*, where *r* is the root canal radius and *h* is the thickness of the sample.

### 2.4. SEM Analysis

One sample was selected randomly from each subgroup and introduced to the characterization of the interfacial area prior to push-out testing to understand whether the nature of the precipitation formed at the interfacial area was amorphous calcium-phosphate leachate. Samples were sputter-coated with 3 –4 nm gold-palladium and examined under a field-emission scanning electron microscope (JEOL, USA) under 2000x magnification with 15 kV voltage and 25 spot size. Irregularities or alterations in the accumulated crystals defined the evaluation criteria of the material-dentin interface, relying on previous studies [20, 23, 27].

### 2.5. Statistical Analysis

The normality of the variables was determined with the Kolmogorov–Smirnov and a one-way ANOVA parametric test followed by Tukey's multiple comparisons for subgroups. All statistical tests were performed by using SPSS version 25.0 (Utah, USA), and the significance level was set at *p*=0.05.

## 3. Results

### 3.1. Bonding Strength

**Figure 6:**
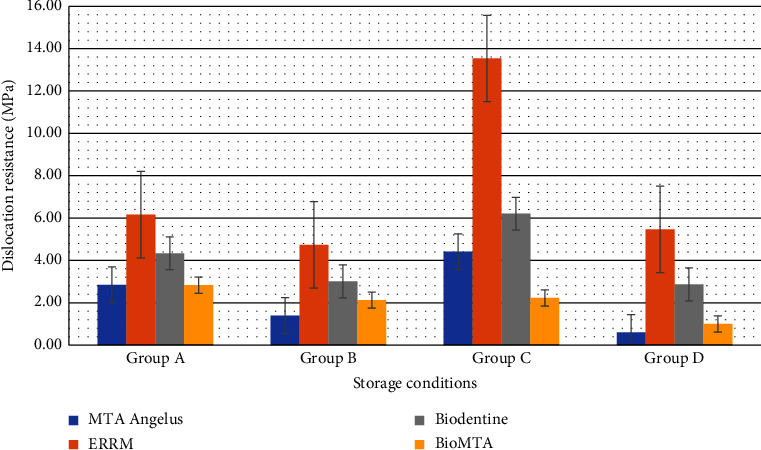
Mean values for dislocation resistance between tested CSC materials. ERRM had the highest values (13,54 ± 5,56 MPa) after exposure to pH 7.2 (group C).

Table 1 shows the mean push-out bonding strength (MPa). ERRM showed the highest bonding resistance regardless of time and storage media compared to MTA, Biodentine, and BioMTA (*p* < 0.05). Biodentine presented a greater bonding resistance compared to MTA and BioMTA in all subgroups (Table 1) (*p* < 0.05). MTA showed greater MPa values than BioMTA; however, the result was not statistically significant (*p* > 0.05).  Figure 6 shows the mean values of the dislocation resistance in all tested materials.

**Figure 7:**
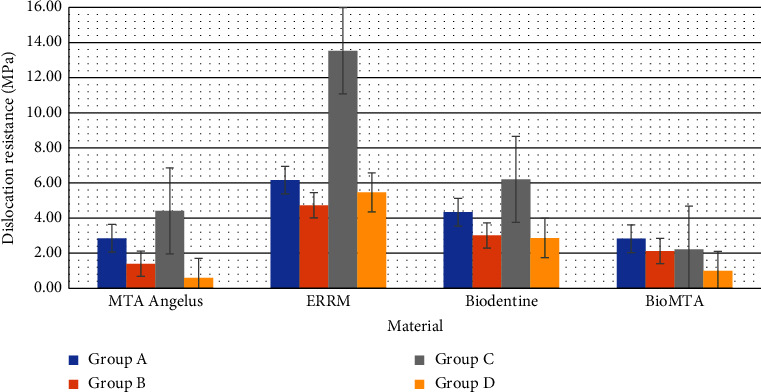
Mean dislocation resistance (MPa) of repair materials after storage in designated subgroups. ERRM, MTA, and Biodentine show the highest MPa values in 34 days of storage in PBS (group C), whereas bioMTA shows the highest values in 4 days of storage in PBS (group A).

The environmental conditions had a significant effect on the bonding resistance of all tested materials. Mean MPa values between the groups according to the storage media and time are shown in Figure 7. Storage in PBS for thirty-four days (group C) showed the highest bonding resistance compared to other subgroups (*p* < 0.05), followed by four days in PBS (group A). Bonding resistance in group A was higher than in group B in all material groups (*p* > 0.05); however, only MTA showed significance (*p* < 0.05). ERRM, Biodentine, and MTA presented significantly higher MPa values when stored in PBS for thirty-four days (group C) compared to storage in four days of PBS or acid (groups A and B). The dislocation resistance resulted in a decrease in group D compared to group B except for ERRM (*p* > 0.05).

### 3.2. SEM Analysis

**Figure 8:**
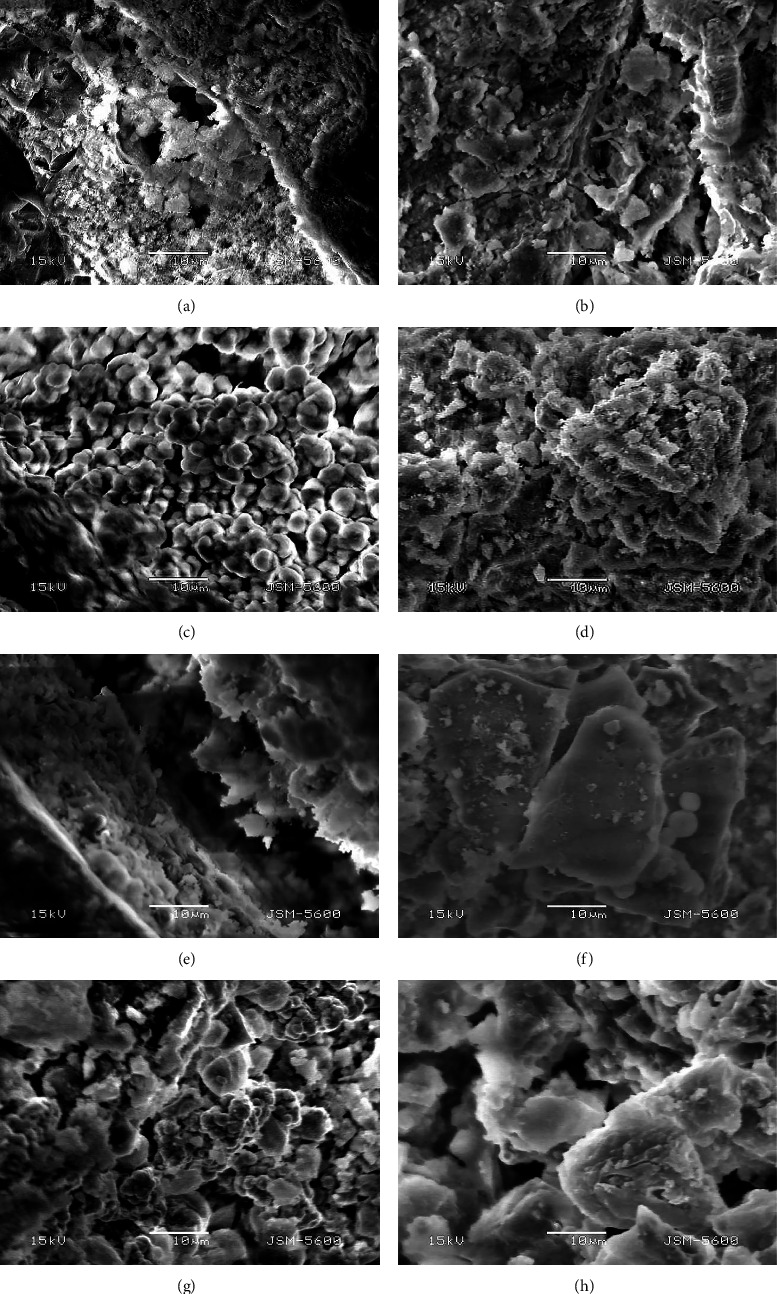
Scanning electron microscopic examination of the interfacial layer for MTA (A and B), ERRM (C and D), Biodentine (E and F), and BioMTA (G and H) (magnification 2000x).

Representative images used to define the interfacial area for each material group are shown as follows. Although the interfacial area of ERRM was tight and almost accumulated entirely with material remnants in all conditions, an acidic challenge created alterations in crystal shape (Figure 8(c)) and recovered after storage in PBS (Figure 8(d)). Biodentine presented large hexagonal crystals following thirty-four days of immersion in PBS (Figure 8(f)). However, acidic conditions created a gap between the material and dentin (Figure 8(e)) and presented a mixed unconnected structure of crystals compared to ERRM. MTA presented a porous formation of crystals after four days in PBS (Figure 8(a)) which were changed into needle-like structures when stored in acid for four days. A thin matrix of crystals covering the interfacial area is visible following storage in PBS for thirty-four days (Figure 8(b)); however, the material failed to recover when stored in PBS after an acidic attack. Similarly, BioMTA presented a globular crystal structure without a tight network of matrices in all groups (Figures 8(g) and 8(h)).

## 4. Discussion

An ideal repair material must withstand various clinical situations as the oral cavity may induce changes in its pH due to acid intake, inflammation, or infection. Therefore, the material used should resist dissolution or breakdown by tissue fluids [28]. The present study simulated a clinical condition by exposing four different endodontic repair materials to acidic and neutral pH for designated periods.

Changes in the pH of host tissues due to a preexistent disease alter the mechanical properties of the CSCs [24, 29]. The mechanical properties include surface hardness [30], tensile strength [31], sealing ability [32], and push-out bonding strength [23]. It is beneficial to investigate the changes in the mechanical properties of the CSCs after immersion in physiological solutions of different pH values [31]. The push-out test determines the interfacial bonding strength by applying a compressive force until the material dislodges from the placement site and is considered the most suitable evaluation method for measuring the bonding resistance of materials to the root dentin [33]. Although the specimen properties, plunger diameter, or alignment of the sample affect the results [34, 35], the test allows standardization of the samples and simulates the clinical forces [36]. Therefore, the dislocation resistance of CSCs in an acidic or alkaline pH has been investigated extensively [24, 26, 37].

When stored in phosphate-containing fluid, the physicochemical interaction between CSCs and dentin results in a chemical bond through an apatite-like structure formation in the interfacial area. A neutral pH is ideal for this formation [38]. Although CSCs may be placed in an environment with low pH, the conditions can improve in time to a desirable state. Therefore, the present study selected acetic acid buffered to pH 4.4 to mimic the compromised environment [26] and compared these findings with improved conditions. Materials placed in PBS for four days showed a higher bonding resistance than in acid storage for four days. This significant decrease in the MPa values after immersion in an acidic condition with pH 4.4 was shown in previously reported results [24, 29]. Specimens stored only in PBS for an extended period significantly increased the dislocation resistance; however, storage in PBS for thirty days following acid exposure was not successful in reversing the compromised bond strength except for ERRM, which contradicted with the findings of Hashem et al. [23]. One possible explanation is that the pH level used in the present study is extreme for the recovery of jeopardized material.

In this study, the highest average bonding strength (13.54 ± 5.56) was observed for ERRM regardless of storage media and time, indicating its adaptation to dentinal walls is better when compared to other material groups, parallel to previously reported results [39]. A study [40] reaching the dislocation of ERRM and MTA in PBS solution for an extended period found that ERRM showed a higher bonding resistance than MTA, parallel to our findings. ERRM is a premixed putty with zirconium oxide added as a filler to enhance specific physical properties [14]. The presence of the zirconia filler may have influenced the higher dislocation resistance of the material. Biodentine also showed better dislocation resistance than MTA and BioMTA but lower MPa values than ERRM. The superiority of Biodentine compared to MTA was shown in previous studies [41]. Biodentine also includes zirconium oxide as a filler agent; however, it requires itself to be mixed with its liquid to control the setting mechanism [13], which requires an application process and may explain the lower MPa values in comparison to ERRM. Also, the setting accelerator and thickening compounds present in the composition of Biodentine might have interfered with the hydration mechanism, especially at a low pH where the crystalline structure is delicate [3]. Further research is necessary to confirm these hypotheses.

Interestingly, according to SEM analysis, both MTA Angelus and BioMTA presented the lowest dislocation resistance in the push-out test with high porosity. This finding may be attributed to these two materials requiring hand-mixing with a specific powder-liquid ratio. The setting reaction for MTA was reported as hydrophilic and required moisture [42] for a certain amount of time. However, the presence of moisture for an adequate time may lead to a moderate increase in the dislocation resistance of MTA [43]. Similarly, our study found a significant increase between PBS groups where the dislocation resistance of MTA increased when in contact with PBS for an extended period (4.42 ± 1.32). However, the MPa values when stored in PBS were low compared to ERRM (13.54 ± 5.56) and Biodentine (6.21 ± 2.20). BioMTA was the material with the lowest dislocation resistance when in contact with PBS. It was reported as a suitable CSC for pulp repair containing hydraulic zirconia complex as a filler [44] with a more extended setting reaction time [45], which may impact the material's solubility, resulting in lower MPa values.

In the present study, MTA had the lowest resistance (1.40 ± 0.63) in acidic pH, whereas ERRM (4.74 ± 2.83) had the highest. Although the crystal structure was affected by low pH resulting in globular, smaller particles, a tightly formed matrix network at the material interface can be seen from SEM images. These findings were consistent with Wang et al. [46], who also reported an increased porosity of materials when subjected to low pH. This difference may be due to the extended exposure period to low pH, which may not be linked to a clinical situation.

BioMTA also presented lower dislocation resistance than ERRM and Biodentine in all storage conditions. In addition, the material showed increased solubility when stored in more extended storage period groups with four days in acid followed by thirty days of PBS, leading to the lowest MPa (1.00 ± 0.23) values in comparison to other materials, indicating the inability to reverse the effects of the compromised environment. Although SEM images presented the accumulation of calcium-silicate particles, the material showed high solubility and low durability when in contact with solutions apart from the pH. Oh et al. [47] showed reduced microhardness for BioMTA when introduced to glycolic and citric acid with large voids and porosities in SEM imaging. However, currently, there are no available data on the dislocation resistance of this material when in contact with acidic and neutral pH in a designated period. Preparation of the material does not include a precise ratio of powder/liquid, which may have influenced the porosities, and a restrained adequate setting before testing. The particle size distribution can also contribute to the sealing ability of calcium-silicate cement according to an average dentinal tubule diameter [48]. Biodentine, constituting particles of increased size for achieving fast setting, was also criticized for the poor cavity adaptation due to this property [13]. Although the average particle size of BioMTA was reported to be 2.6 *µ*m [49], our study revealed reduced strength and increased porosity compared to Biodentine. Further research is necessary to confirm these findings.

One of the drawbacks of the present study was creating optimal clinical conditions. Although a standardized model with a 1.3 mm lumen size was used for the reproducibility of the analysis, a perforation cavity may vary in size and location. It may also be affected through contamination with tissue fluids or endodontic irrigants [29, 46]. Aggarwal et al. [37] used perforation cavities prepared in extracted teeth to investigate blood contamination, similar to Hansen et al. [50], who also used extracted teeth to simulate the resorptive canal defects. A permanent restoration affects the outcome of the treatment. Palma et al. [51] presented that placement of a delayed restoration on top of MTA is better due to its lower shear bonding values, whereas an immediate restoration can be applied on Biodentine.

The setting time of the cement may influence the results. In the present study, following mixing and applying the materials into specimen lumens, the samples were immediately immersed in storage solutions which may have influenced the setting time and, eventually, the dislocation resistance. Although a precise setting time for ERRM is not available, it was previously reported that while 48 hours is not enough for a complete set, 36 hours is adequate for MTA [52]. Moreover, physical attempts can be a significant factor in the dislocation resistance of endodontic repair materials. Nekoofar et al. [53] described the impact of condensation pressure on the physical properties of MTA and presented that higher pressures resulted in low surface hardness. Mixing techniques were also an influencing factor for the dislocation resistance [54, 55]. The method chosen for mixing is essential to obtain the desired interaction of powder and liquid for the material setting. When the powder particles cannot be hydrated sufficiently, the material's durability could be reduced. Therefore, using a standardized mixture is important [49]. Accordingly, premixed types of cement gave favorable physicochemical results in terms of bonding strength and sealing ability [56, 57] by the results of our study. However, future studies are required to validate this outcome.

## 5. Conclusion

According to the results of this study, ERRM showed a higher dislocation resistance among all test materials. BioMTA, a novel calcium-silicate cement, presented increased porosity and lower durability in given conditions. Storage in PBS for a more extended period significantly improved the bond strength but failed in reversing the effects of the comprised environment caused by the low pH.

## Figures and Tables

**Table 1:** The dislocation resistance (MPa) regarding storage time and media is given in means ± SD.

	MTA				ERRM				Biodentine				BioMTA			
	PBS		Acid		PBS		Acid		PBS		Acid		PBS		Acid	
Material time	Mean	SD	Mean	SD	Mean	SD	Mean	SD	Mean	SD	Mean	SD	Mean	SD	Mean	SD
4 days	2.85^Bb^	1.57	1.40^Cd^	0.63	6.17^Ba^	3.19	4.74^Bc^	2.83	4.34^Ba^	1.17	3.01^Bd^	0.55	2.83^Ab^	0.61	2.12^Ad^	0.83
34 days	4.42^Afg^	1.32	0.60^Cj^	0.34	13.54^Ae^	5.56	5.47^Bh^	2.95	6.21^Afi^	2.20	2.87^Ci^	0.43	2.22^Ag^	0.65	1.00^Bj^	0.23
*p* value	**0.005**		0.28		**<0.001**		0.96		**0.005**		0.99		0.09		**<0.001**	

Uppercase superscript letters indicate significance in each row; lowercase superscript letters indicate significance in each column.

## Data Availability

The principal investigator, Beliz Ozel, has full access to all data and holds responsibility for the integrity and accuracy of data analysis.
